# Prevalence of *Wolbachia* in natural sand fly (diptera: psychodidae) populations from Türkiye and its potential role in mitochondrial divergence

**DOI:** 10.1186/s13071-025-07157-4

**Published:** 2025-12-04

**Authors:** Ayda Yilmaz, Ozge Erisoz Kasap

**Affiliations:** 1 Department of Biology, Ecology Section, Faculty of Science, VERG Laboratories, Hacettepe University, Ankara, Türkiye; 2 Graduate School of Science and Engineering, Hacettepe University, Ankara, Türkiye

**Keywords:** Phlebotomine, Endosymbionts, MLST, Selective sweep

## Abstract

**Background:**

Phlebotomine sand flies are vectors of various pathogens, most notably *Leishmania* spp. Symbiotic bacteria have recently gained considerable attention owing to their effects on hosts and on other organisms co-infecting the same host. In this study, we investigated the natural *Wolbachia* infection status of sand fly taxa distributed in Türkiye and examined its potential role in driving the deep mitochondrial divergence observed within certain taxa.

**Methods:**

We analysed 858 sand fly specimens, mostly collected between 2005 and 2016, with additional samples obtained in 2023. Specimens were morphologically identified, and the mitochondrial *cox1* gene was sequenced for DNA barcoding. For selected taxa showing marked mitochondrial divergence, species delimitation methods were applied, and genetic diversity indices and neutrality tests were calculated. *Wolbachia* infection was detected via PCR amplification of the *wsp* gene, and strain diversity was characterised using multilocus sequence typing (MLST) of five housekeeping genes. Logistic regression was used to evaluate associations between infection status and mitochondrial lineage, sex or collection period.

**Results:**

*Wolbachia* infection was detected in 16.67% of specimens, occurring exclusively in *Phlebotomus papatasi*, *Ph. major* s.l., *Ph. tobbi*, *Ph. economidesi* and *Sergentomyia minuta*. Analyses of *wsp* and MLST data identified all sequences as belonging to Supergroup A, with multiple strains present within and across host taxa. Infection among the five *Ph. major* s.l. lineages delineated by species delimitation was significantly associated with lineage, with lineages 3–5 showing a higher probability of infection. The reduced haplotype and nucleotide diversity, along with a significant negative deviation from neutrality observed in lineage 5, suggest a selective sweep likely driven by *Wolbachia* infection.

**Conclusions:**

This study represents the first comprehensive screening of *Wolbachia* infection in sand fly taxa distributed across Türkiye, during which several novel *Wolbachia* strains were identified. Our findings suggest a potential role of *Wolbachia* infection in driving lineage differentiation within certain sand fly taxa. However, further detailed investigations are required to elucidate the mechanisms by which *Wolbachia* influences sand fly diversification and to assess the broader epidemiological implications related to sand fly-borne diseases (SFBDs).

**Graphical Abstract:**

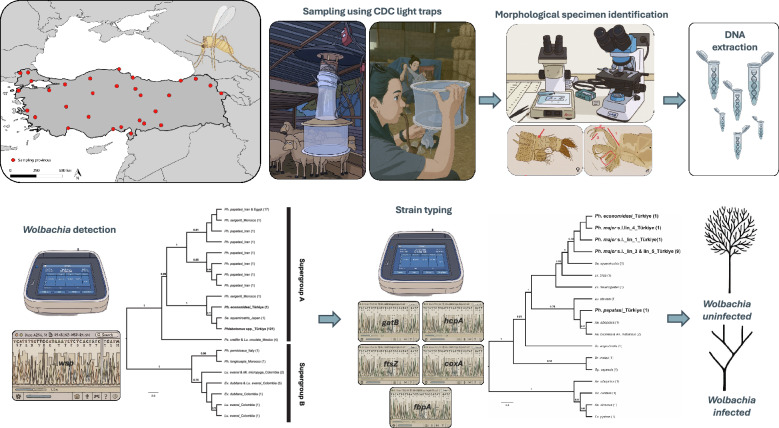

**Supplementary Information:**

The online version contains supplementary material available at 10.1186/s13071-025-07157-4.

## Background

Phlebotomine sand flies are of considerable medical and veterinary importance owing to their role in the transmission of *Leishmania* spp. parasites, various arboviruses and bacteria such as *Bartonella* spp. The distribution of more than 1000 identified sand fly species is primarily driven by species-specific microclimatic conditions; nevertheless, their global range extends from approximately 50 °N to 40 °S latitude, although they are absent from New Zealand and most Pacific islands [1, 2]. Among the diseases transmitted by sand flies, leishmaniasis is listed as one of the World Health Organization (WHO)’s Neglected Tropical Diseases (NTDs), with an estimated 50,000–90,000 new cases of its potentially fatal visceral form (VL) and up to one million cases of the cutaneous form (CL) occurring annually. The disease burden is disproportionately high in South Asia, East Africa, the Mediterranean region, South America and Central Asia [3]. For the past two decades, significant progress has been made in understanding sand fly biology, ecology and immune responses to *Leishmania* infection. However, gaps regarding knowledge of the behaviour, population structure and natural parasite–vector–host interactions remain to be filled. These gaps continue to limit the development of effective sand fly or leishmaniasis control strategies, contributing to the burden of leishmaniasis and its classification as a neglected tropical disease [4].

The facultative symbiotic interaction between the Gram-negative *Wolbachia* bacterium and its arthropod hosts offers a promising biological tool for the design of integrated vector management strategies. Dating back approximately 100 million years, this interaction provides several metabolic benefits to both the host and the bacterium [5, 6]. Infecting approximately half of all terrestrial arthropod species, maternally inherited *Wolbachia* can affect host population dynamics by inducing cytoplasmic incompatibility (CI) between the sperm and eggs of conspecifics [7], male killing [8], feminisation [9] and parthenogenesis [10]. In this context, CI induced by *Wolbachia* infection has proven to be a valuable tool for controlling vector population densities by reducing the fecundity or fitness of adult insects. This approach primarily relies on releasing *Wolbachia*-infected male specimens into the natural environment. If the wild females are either uninfected or infected with an incompatible *Wolbachia* strain, fertilisation fails owing to cytoplasmic incompatibility (see [11] for review). It has also been well documented that *Wolbachia* acts as a barrier to pathogens by interfering with their replication within the host, thereby suppressing disease transmission [12–14]. Owing to the manipulative effects of *Wolbachia* on its hosts – most frequently in mosquitoes – an increasing number of laboratory and field studies on *Wolbachia*–host and *Wolbachia*–pathogen interactions have been conducted aiming to enhance the success of *Wolbachia*-based population replacement and suppression efforts in both vector control and disease management programmes [15, 16].

*Wolbachia* infection in natural sand fly populations has been reported in several studies conducted across both the New and Old Worlds, with infection rates and *Wolbachia* strains varying within and between species depending on geographic location [17–20]. In contrast to mosquitoes, a substantial lack of information regarding the interaction between *Wolbachia* and the pathogens transmitted by sand flies remains. Another overlooked aspect is the potential role of *Wolbachia* in driving mitochondrial diversification in sand flies through genetic hitchhiking resulting from maternal inheritance. Although this phenomenon is documented in several insect taxa [21–23], its impact on sand flies has received limited attention. This may have significant implications for both sand fly taxonomy and the epidemiology of sand fly-borne diseases (SFBDs).

Leishmaniasis has been endemic in Türkiye, with its epidemiological pattern shifting in response to changing environmental and social conditions. These changes have led to the emergence of new transmission cycles involving newly recorded parasite species, hybrid forms, and potential vectors and reservoirs (see [24] for a review). The number of formally described sand fly species in Türkiye is 28. However, comprehensive surveillance and molecular barcoding efforts have revealed the presence of 33 molecularly distinct taxonomic units, with several mitochondrial lineages of certain taxa distributed widely across the country, occurring either sympatrically or allopatrically [25]. To date, only a few attempts have been made to screen for *Wolbachia* infections in natural sand fly populations in Türkiye [26, 27], and these studies were confined to limited geographical areas. Furthermore, no data are currently available on the potential effects of endosymbiotic interactions on the cryptic diversification of sand flies in the country.

In this study, we aimed to determine *Wolbachia* infection rates among natural sand fly populations representing most of the taxa distributed in Türkiye; assess whether *Wolbachia* prevalence varies across a wide temporal and geographical range; characterise *Wolbachia* strain diversity; and evaluate the potential impact of *Wolbachia* infections on the mitochondrial diversification observed within certain taxa.

## Methods

### Sand fly specimens

**Fig. 1:**
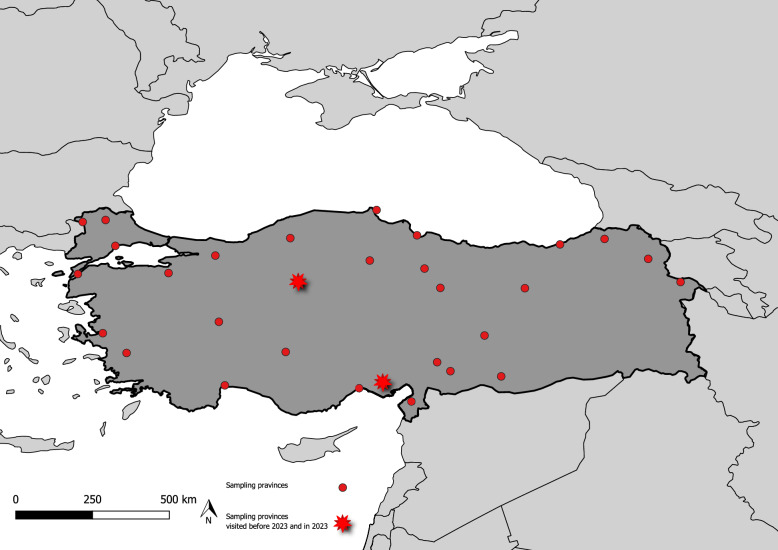
Map showing the provinces where sand fly collections were conducted in 2005 and 2023, indicating sampling activities both prior to and during fieldwork in 2023

A total of 858 sand fly specimens were screened for *Wolbachia* infection. Most of these specimens were collected during field activities conducted between 2005 and 2016 and are part of our laboratory’s archived collection. These specimens had already been morphologically identified and barcoded in a previous study [25]. A small subset (*n* = 31) was collected more recently, during fieldwork carried out in 2023 (Fig. 1). Specimens collected prior to 2023 were stored in 90% ethanol, while those collected in 2023 were stored in 70% ethanol at –40 °C. Detailed information on the sand fly taxa and the mitochondrial lineages included in this study – previously inferred based on our earlier findings [25] – is summarised in Table 1.

**Table 1:** *Wolbachia* infection prevalence among sand flies by taxa and sex

Subgenus	Taxa	Female	Male	Total	Infected females (%)	Infected males (%)	Infected total (%)
*Phlebotomus*	*Ph. papatasi*	13	21	34	3 (23.08)	4 (19.05)	7 (20.59)
*Artemievus*	Ph. alexandri *	8	21	29	0 (0.00)	0 (0.00)	0 (0.00)
*Paraphlebotomus*	*Ph. caucasicus*	0	1	1	x	0 (0.00)	0 (0.00)
	*Ph. jacusieli*	3	2	5	0 (0.00)	0 (0.00)	0 (0.00)
	*Ph. sergenti* s.l. **	31	26	57	0 (0.00)	0 (0.00)	0 (0.00)
*Larroussius*	*Ph. kandelakii* s.l. **	17	46	63	0 (0.00)	0 (0.00)	0 (0.00)
	*Ph. major* s.l. ***	46	166	212	36 (78.26)	97 (58.43)	133 (62.74)
	*Ph. perfiliewi* s.l	45	81	126	0 (0.00)	0 (0.00)	0 (0.00)
	*Ph. tobbi*	50	71	121	1 (2.00)	0 (0.00)	1 (0.83)
*Adlerius*	*Ph. balcanicus*	4	5	9	0 (0.00)	0 (0.00)	0 (0.00)
	*Ph. brevis*	2	1	3	0 (0.00)	0 (0.00)	0 (0.00)
	*Ph. halepensis*	25	26	51	0 (0.00)	0 (0.00)	0 (0.00)
	*Ph. kyreniae*	2	5	7	0 (0.00)	0 (0.00)	0 (0.00)
	*Ph. simici*	27	22	49	0 (0.00)	0 (0.00)	0 (0.00)
	*Adlerius* sp.	8	0	8	0 (0.00)	x	0 (0.00)
*Transphlebotomus*	*Ph. anatolicus*	3	3	6	0 (0.00)	0 (0.00)	0 (0.00)
	*Ph. economidesi*	1	0	1	1 (100.00)	x	1 (100.00)
	*Ph. killicki*	1	6	7	0 (0.00)	0 (0.00)	0 (0.00)
*Sergentomyia*	Se. dentata*	22	14	36	0 (0.00)	0 (0.00)	0 (0.00)
	Se. minuta**	22	11	33	0 (0.00)	1 (9.09)	1 (3.03)
	Total	**330**	**528**	**858**	**41 (12.42)**	**102 (19.32)**	**143 (16.67)**

Taxa marked with one asterisk (*) have two mitochondrial lineages, two asterisks (**) indicate three lineages and three asterisks (***) indicate five lineages. Taxa without an asterisk have a single lineage, based on Kasap et al. (2019)

### Sand fly specimen identification and barcoding

A total of 25 *Phlebotomus major* s.l. Annandale, 1910 specimens were recently collected from two provinces (Adana and Ankara) where this taxon had previously been detected, in order to determine whether the same lineages are still present and to compare their *Wolbachia* infection status across different sampling periods. In addition, seven *Ph. perfiliewi* s.l. Parrot, 1939 specimens, collected from a previously unsurveyed province (Corum), were also barcoded (Fig. 1). The head and the last two to three abdominal segments of these specimens were dissected and slide-mounted for morphological identification, while the remaining abdominal tissue was used for DNA extraction. Morphological identification was performed using the PhlebKeyTool [28]. DNA was extracted using the Qiagen DNeasy Blood and Tissue Kit (Qiagen, Hilden, Germany). The mitochondrial cytochrome *c* oxidase subunit I (*cox1*) gene was then amplified using the primers LCO1490 and HCO2198 [29] as described previously [30].

### *Wolbachia *detection and strain characterisation

*Wolbachia* prevalence was estimated by amplifying a ~340 bp fragment of the *wsp* gene [26], and strain diversity was further characterized using multilocus sequence typing (MLST) based on five housekeeping genes: *gatB*, *coxA*, *hcpA*, *ftsZ* and *fbpA* [31]. For each PCR amplification, *Drosophila melanogaster* Meigen, 1830 (DGRP-RAL-100 genotype) was used as a positive control, and a no-template reaction was included as a negative control. Primer sequences and optimised thermal conditions for each amplification reaction are provided in Additional file 1.

### DNA sequencing and sequence analysis

Bidirectional sequencing was carried out using the same primers for each amplified gene (BMLabosis, Ankara, Türkiye). All sequences were checked and edited using BioEdit (v2.7.5) and Chromas (v2.6.4). All positive *wsp* amplification products were subjected to sequencing to determine their supergroup classification, while only a subset of specimens had their MLST loci sequenced for strain identification. Unique *wsp* haplotypes obtained in this study, along with other *wsp* sequences previously generated for New and Old World sand fly species and deposited in GenBank [32], were used to perform a maximum likelihood (ML) analysis under the best-fit nucleotide substitution model, with 1000 bootstrap replicates, using MEGA6 [33]. The concatenated MLST sequences were queried against the *Wolbachia* MLST database [34] to identify their allelic profiles. A data set was generated using the unique MLST haplotypes and concatenated MLST sequences obtained from PupMLST and GenBank, representing different *Wolbachia* supergroups. Using these dataset, a ML tree was constructed under the best-fit substitution model, with 1000 bootstrap replicates, in MEGA6.

The *cox1* sequences obtained from recently collected specimens were compared with those stored in our in-house reference barcode library for Turkish sand flies (GenBank accession numbers are available in [25]. Lineage assignment of these specimens was carried out using the assemble species by automatic partitioning (ASAP) method [35], implemented via the SPART Explorer web platform [36]. The best-scoring partition file produced by ASAP was then used to generate a haplotype network using the TCS algorithm implemented in the Hapsolutley tool [37]. Diversity estimates for each lineage, along with neutrality test statistics (Fu’s Fs [38] and Tajima’s D [39]), were calculated using DnaSP (v5.10).

The newly generated sequences for sand fly *cox1*, *Wolbachia wsp*, and MLST loci have been submitted to GenBank under accession numbers PX507678-PX507709 and PX559865-PX559937.

### Statistical analysis

In order to overcome the issue of ‘complete separation’ caused by rare outcomes and limited data, Firth’s penalised binary logistic regression model [40] was used to investigate the relationship between *Wolbachia* infection status, sand fly mitochondrial lineages and the sex of the specimens. The same approach was also used to evaluate whether infection status varied across different time periods (i.e. specimens collected before 2023 and those collected in 2023) using the ‘logistf’ package implemented in Rstudio [41] 42.

## Results

### Wolbachia* prevalence among sand fly taxa*

The overall *Wolbachia* infection rate among the 858 retrospectively and recently collected sand fly specimens, representing 20 taxa, was 16.67% (143/858). Infection prevalence was slightly higher in males (19.32%, 102/528) than in females (12.42%, 41/330). Not including *Ph. economidesi*, for which the only screened specimen was infected (100%), the highest *Wolbachia* infection rate was recorded in *Ph. major* s.l., with 62.74% of specimens (133/212) testing positive. *Ph. papatasi* (Scopoli, 1786) showed the second-highest prevalence at 20.59% (7/34). A single infected specimen was also detected in *Ph. tobbi* Adler, Theodor and Lourie, 1930 and *Sergentomyia minuta* (Rondani, 1843). All remaining sand fly taxa tested negative for *Wolbachia* based on *wsp* gene amplification (Table 1). PCR assay reliability was confirmed using *Drosophila melanogaster* as a positive control and including negative controls, which consistently showed no amplification, validating the *Wolbachia* detection results.

## *Mitochondrial lineages of *Ph. major *s.l. and their infection status*

**Fig. 2:**
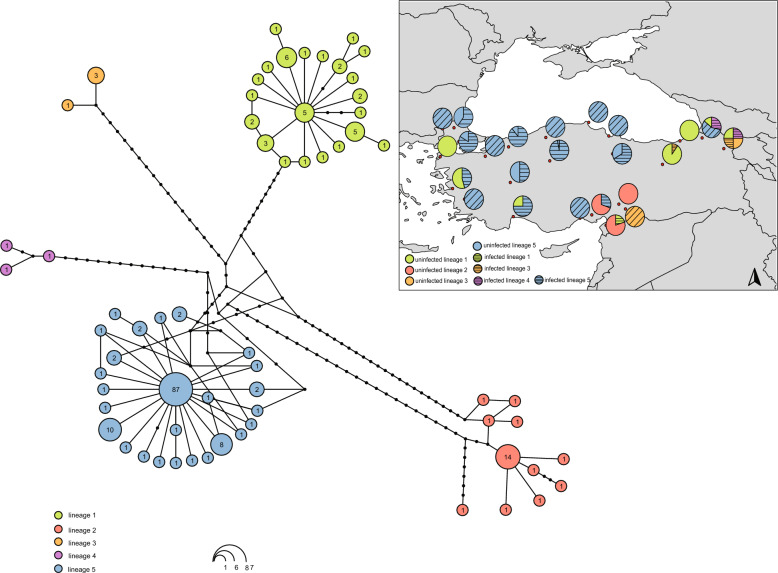
Combined results of TCS haplotype network analysis and ASAP species delimitation for the 202 *Ph. major* s.l. specimens analysed. The map shows the geographic distribution of different lineages, represented by pie charts with distinct colours for each lineage. The dashed segments within the pie charts indicate the proportion of specimens infected with *Wolbachia* in each lineage

In concordance with previously reported findings [25], the inclusion of 25 newly generated *cox1* sequences in the previously published dataset confirmed the existence of five distinct lineages within the *Phlebotomus major* s.l. complex, some of which occur sympatrically in Türkiye, based on the results of both the ASAP species delimitation method and TCS haplotype network analysis. *Wolbachia* screening revealed that, out of 40 specimens belonging to the first lineage (formally described as *Ph. neglectus* Tonnoir, 1921), only a single specimen was positive for *Wolbachia*, while all *Ph. syriacus* Adler and Theodor, 1931 (lineage 2) specimens (*n* = 24) tested negative. Among the not yet formally described members of the complex, three out of four specimens assigned to lineage 3, all specimens in lineage 4 (3/3), and 120 out of 132 specimens in lineage 5 were found to be infected (Fig. 2).

Six specimens collected in 2023, from a locality (Adana) where only *Ph. syriacus* had previously been recorded, were all infected and assigned to lineage 5, whereas all uninfected specimens recently collected from the same site were identified as *Ph. syriacus*. Lineage assignment of the newly collected specimens from a second locality (Ankara) – where lineage 5 had been recorded in the past – remained consistent, with 10 out of 11 specimens again testing positive (Fig. 2).

**Fig. 3:**
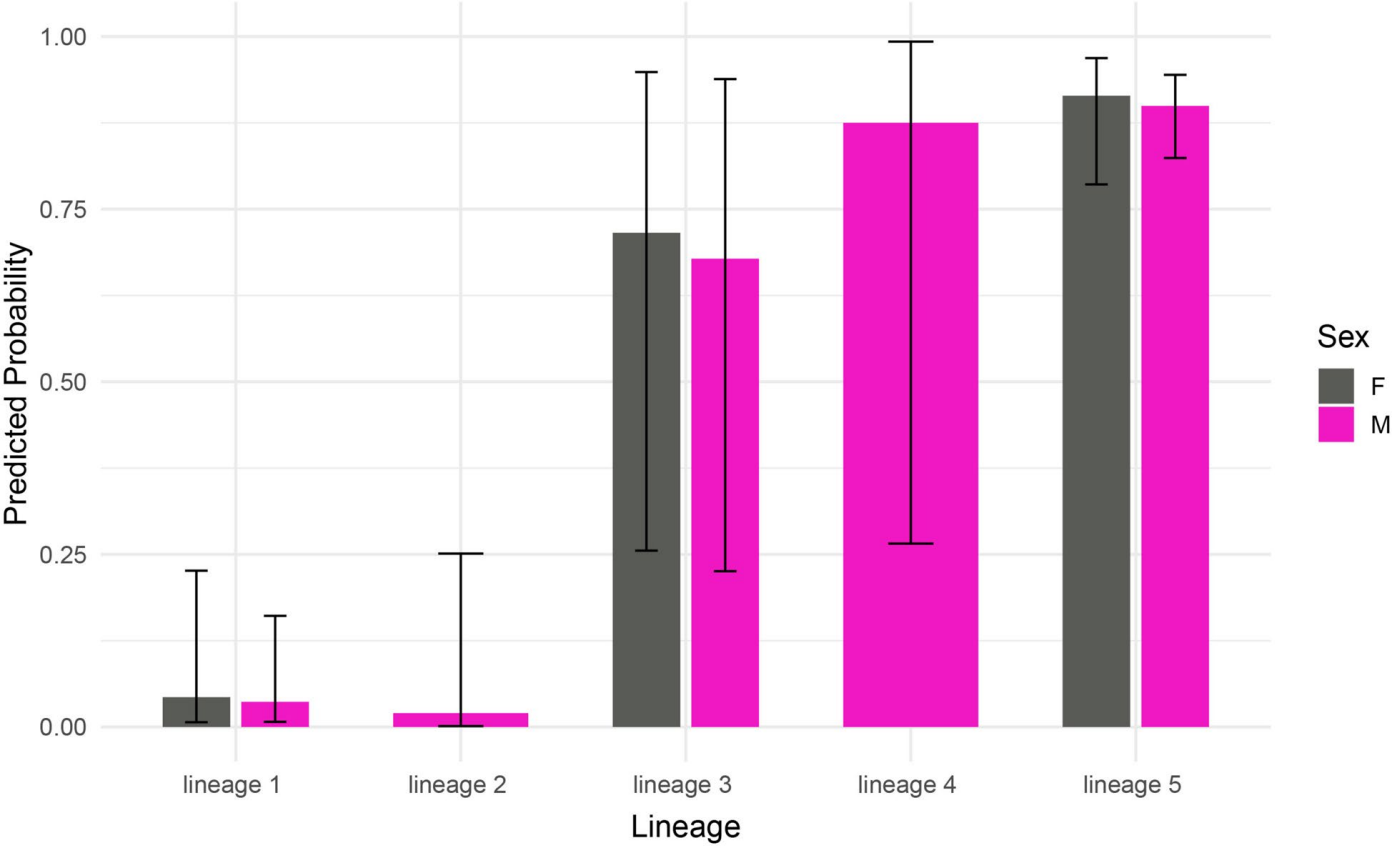
Probability of *Wolbachia* infection across mitochondrial lineages and sex, estimated using Firth’s penalised logistic regression model. Error bars represent upper and lower 95% confidence intervals

Firth’s logistic regression results indicated that lineages 3, 4 and 5 had significantly higher odds of *Wolbachia* infection compared with the reference lineage (lineage 1, *Ph. neglectus*), with odds ratios (OR) of 55.8 (95% CI: 5.50–1067.13), 185.3 (95% CI: 11.36–30,295.38) and 236.6 (95% CI: 55.98–2216.93), respectively (*P* < 0.001). In contrast, the odds of infection in lineage 2 (*Ph. syriacus*) were not significantly different from *Ph. neglectus* (OR = 0.54; 95% CI: 0.004–10.61; *P* = 0.698). Similarly, sex was not significantly associated with infection status (OR for males = 0.84; 95% CI: 0.22–2.69; *P* = 0.774) (Fig. 3). A separate model, constructed using only specimens from lineages 2 (*Ph. syriacus*) and 5 collected at the same sites across different time periods, revealed temporal stability in *Wolbachia* infection within these lineages, with no significant effect of time on infection status (*p* = 0.425).

In addition to the *Ph. major* s.l. complex, seven newly barcoded *Ph. perfiliewi* s.l. specimens collected from a previously unsurveyed province clustered within the single lineage previously defined for this taxon, confirming its broad geographic distribution [25] (Additional file 2). Both the newly sequenced and previously available *Ph. perfiliewi* s.l*.* specimens were negative for *Wolbachia* infection.

## *Wolbachia* strain characterisation

**Fig. 4:**
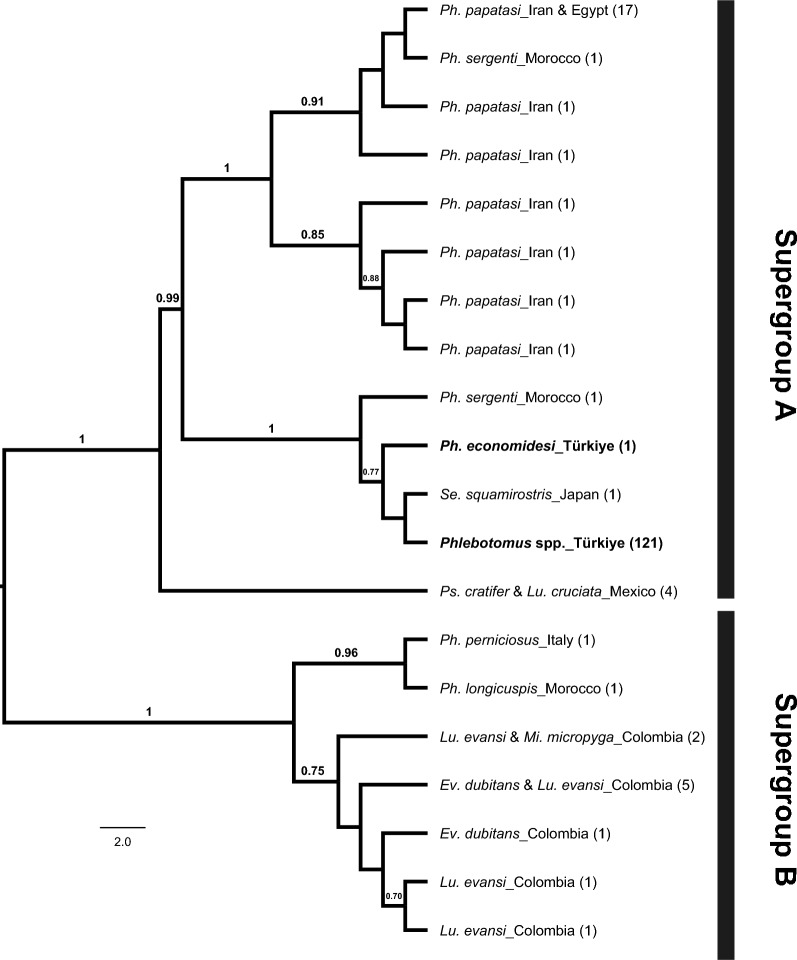
Maximum likelihood phylogenetic tree based on *wsp* haplotypes obtained in this study (in bold) and those retrieved from GenBank. Numbers in parentheses indicate the number of specimens assigned to each corresponding haplotype. Bars represent the corresponding *Wolbachia* supergroups. Only bootstrap values greater than 70% are shown

Of the 143 *Wolbachia*-infected specimens, 121 (83.92%) yielded reliable *wsp* gene sequences from *Ph. papatasi*, *Ph. major* s.l., *Ph. tobbi*, *Ph. economidesi* and *Se. dentata*. Only two unique *wsp* haplotypes were identified, indicating low haplotype diversity (Hd = 0.0165). One haplotype was exclusive to the *Ph. economidesi* specimen, while the other was shared among the remaining taxa. Maximum likelihood (ML) analysis, conducted using these haplotypes alongside others previously reported from sand fly taxa in various countries, and under the assumptions of the Tamura 3-parameter substitution model with Gamma distribution, revealed that the Turkish *wsp* haplotypes clustered within supergroup A. These haplotypes showed close phylogenetic relationships with those from *Ph. sergenti* Parrot, 1917 in Morocco and *Se. squamirostris* (Newstead, 1923) in Japan (Fig. 4). Detailed information on the *wsp* haplotypes obtained in this study and those retrieved from GenBank is provided in Additional file 3.

We were unable to amplify the MLST loci for *Ph. tobbi* and *Se. minuta*. In contrast, successful amplification of all MLST loci was achieved for the subsampled *Ph. papatasi* specimens (*n* = 2) and the single infected *Ph. economidesi* specimen. Among the *Ph. major* s.l. lineages, complete MLST profiles were obtained from the only infected specimen in lineage 1 (*Ph. neglectus*), two out of four specimens in lineage 3 and one out of three specimens in lineage 4. In lineage 4, amplification failed for the *fbpA* and *hcpA* loci in one specimen, and for the *hcpA* locus in the other. Complete allelic profiles were successfully obtained for six individuals from lineage 5, which were selected from different provinces as a subsample owing to limited resources (Table 2).

**Table 2:** Exact allelic profiles obtained from PubMLST of *Wolbachia*-infected sand fly specimens based on *wsp* and five MLST loci with their corresponding specimen code, taxa and mitochondrial lineage

Sand fly taxa	Lineage	Specimen code	*coxA*	*fbpA*	*ftsZ*	*gatB*	*hcpA*
*Ph. papatasi*	n.a	KDIS55	15	17	6	46	23* (^360^A to ^360^G)
		KLM2.1	2	97	31* (^60^ T to ^60^C)	22	42* (^150^G to ^150^C; ^378^T to ^378^C)
*Ph. economidesi*	n.a	BLD5.49	9	15	17	6	46* (^355^G to A^355^; ^360^A to ^360^G)
*Ph. major* s.l	1	SEB 19	15	17	6	46	23* (^87^C to ^87^T; ^386^A to ^386^G)
	3	YUVA1.15	15	17	6	46	23* (^360^A to ^360^G)
		KARA14.104	15	17	6	46	23* (^360^A to ^360^G)
	4	CML3.2	15* (^54^C to ^54^ T)	17* (^244^G to ^244^A)	6	46	23* (^265^G to ^265^A; ^360^A to ^360^G;,^425^ G to ^425^A)
		KOT78.25	15* (^54^C to ^54^ T)	x	6	46	x
		CML3.8	15* (^54^C to ^54^ T)	17* (^244^G to ^244^A)	6	46	x
	5	YKP1.8	15	17	6	46	23* (^360^A to ^360^G)
		SARI2	15	17	6	46	23* (^360^A to ^360^G)
		ZER2.32	15	17	6	46	23* (^360^A to ^360^G)
		GUD4.33	15	17	6	46	23* (^360^A to ^360^G)
		SFR41	15	17	6	46	23* (^360^A to ^360^G)
		KOP6	15	17	6	46	23* (^360^A to ^360^G)

n.a.: sand fly taxa represented by one mitochondrial lineage. x: MLST loci that were not successfully amplified and sequenced. Asterisk (*) indicate the closest hit obtained from the PubMLST. The position of nucleotide differences compared with the closest allele was shown in parentheses

The concatenated MLST sequences obtained from *Ph. papatasi* (*n* = 2), *Ph. major* s.l. (*n* = 10), and *Ph. economidesi* (*n* = 1) were 2079 bp in length. Five distinct haplotypes were identified (Hd = 0.5385). One *Ph. papatasi* specimen and all *Ph. major* s.l. specimens from lineages 3 and 5 shared the same haplotype, suggesting infection with the same *Wolbachia* sequence type. In contrast, the second *Ph. papatasi* specimen, *Ph. economidesi* and *Ph. major* s.l. specimens from lineages 1 (*Ph. neglectus*) and four each exhibited unique allelic profiles (Table 2). No exact sequence type (ST) matches were found in the MLST database for any of the concatenated sequences obtained in this study.

**Fig. 5:**
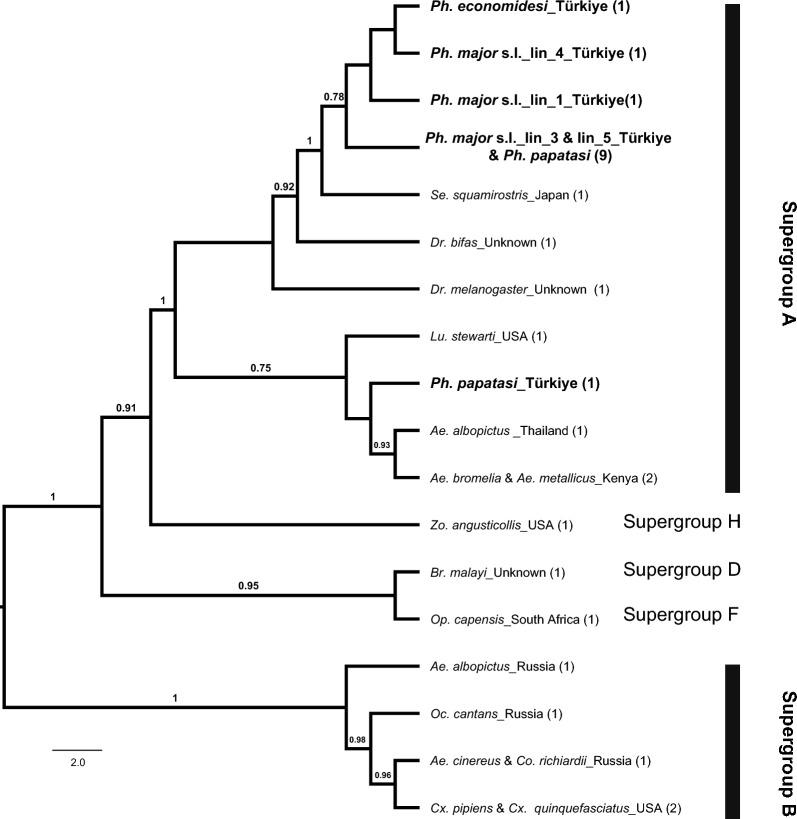
Maximum likelihood phylogenetic tree based on MLST haplotypes obtained in this study (in bold) and those retrieved from GenBank and PubMLST. Numbers in parentheses indicate the number of specimens assigned to each corresponding haplotype. Bars represent the corresponding *Wolbachia* supergroups. Only bootstrap values greater than 70% are shown

Consistent with the results from the *wsp* dataset, maximum likelihood (ML) analysis based on the unique MLST haplotypes – combined with reference sequences from the PubMLST database and GenBank representing various *Wolbachia* supergroups – showed that infected sand fly specimens from Türkiye clustered within supergroup A. According to the ML tree constructed using the Tamura 3-parameter substitution model with a Gamma distribution, the unique concatenated MLST sequence from one *Ph. papatasi* specimen was more closely related to the New World sand fly *Lutzomyia stewarti* (Mangabeira Fo and Galindo, 1944), whereas the remaining Turkish MLST haplotypes were more closely related to the Old World sand fly *Sergentomyia squamirostris* (Fig. 5). No evidence of recombination was detected among the five MLST loci based on individual phylogenetic analyses (Additional file 4).

## Comparative genetic diversity and neutrality among *Ph. major* s.l. mitochondrial lineages

To evaluate the effect of *Wolbachia* infection on genetic diversification within *Phlebotomus major* s.l., we analysed a 606 bp fragment of the *cox1* gene from 203 specimens, which represent 95.75% of all *Ph. major* s.l. specimens analysed, encompassing five mitochondrial lineages. Across all 203 specimens, 64 haplotypes were identified (Hd = 0.807, *π* = 0.02543). Haplotype and nucleotide diversity values recorded for lineages 1 (*Ph. neglectus*) and 2 (*Ph. syriacus*) – both largely uninfected (1/40) or entirely uninfected (none) – were considerably higher than those observed in lineage 5, which showed high infection prevalence (120/132). The significantly negative Fu’s Fs and Tajima’s D values obtained for all three lineages may suggest that these lineages have undergone recent demographic expansion or are subject to purifying selection. However, in contrast to lineage 2, which is restricted to the southeastern region of Türkiye and represented by 24 sequences assigned to 11 haplotypes (Hd = 0.670), and lineage 1, which has a relatively limited and patchy distribution in the northeastern and western localities with 40 specimens assigned in 22 haplotypes (Hd = 0.947), only 26 haplotypes were detected among the 132 specimens of lineage 5 (Hd = 0.558), which is widely distributed and was sampled from nearly all sites where the species complex occurs. The majority of these specimens (*n* = 87) were represented by a single haplotype, regardless of their geographic origin. This markedly low haplotype diversity (Hd) in lineage 5, accompanied by a low nucleotide diversity (*π* = 0.00126), may indicate a *Wolbachia*-induced selective sweep, leading to the fixation of a small number of mitochondrial haplotypes within this lineage. The effect of *Wolbachia* infection on lineages 3 and 4 remains unclear owing to the limited sample sizes available for these lineages (*n* = 4 and *n* = 3, respectively) (Table 3).

**Table 3:** Genetic diversity and neutrality test results in relation to *Wolbachia* infection across divergent *Phlebotomus major* s.l. lineages (*P* < 0.05*, *P* < 0.02**, *P* < 0.001***)

*Ph. major* s.l	lineage_1	lineage_2	lineage_3	lineage_4	lineage_5
No. of sequences (N)	40	24	4	3	132
No. of polymorphic sites (S)	22	21	2	4	25
Average no. of nucleotide differences (k)	2.226	2.370	1.000	2.667	0.766
Nucleotide diversity (π)	0.00367	0.00391	0.00165	0.00440	0.00126
No. of haplotypes (h)	22	11	2	3	26
Haplotype diversity (Hd)	0.947	0.670	0.500	1.000	0.558
Observed Haplotype diversity (Ho)	0.550	0.458	0.500	1.000	0.197
Fu's Fs	−19.764 ***	−4.235 *	1.099	NA	−36.337 ***
Tajima's D	−1.904 *	−2.120 *	–0.710	NA	−2.434 **

## Discussion

Among the 28 sand fly taxa formally reported from Türkiye, six taxa – *Ph. alexandri*, *Ph. sergenti*, *Ph. kandelakii* s.l. Schurenkova, 1929, *Ph. major* s.l., *Se. dentata* and *Se. minuta* – have previously been shown to harbour 2–5 mitochondrial lineages based on a comprehensive *cox1* barcoding effort [25]. In the present study, *Wolbachia* prevalence was assessed across the majority of the sand fly fauna of Türkiye, including taxa with a single mitochondrial lineage, to better understand its distribution and potential role in sand fly diversification.

The overall *Wolbachia* infection rate was 16.67%; however, this was largely driven by *Ph. major s.l.*, which accounted for 133 of the 143 infected specimens (93%) and represented 24.7% of all screened specimens (212/858). Most other taxa tested negative regardless of their mitochondrial lineage diversity. All species belonging to the subgenera *Artemievus* Depaquit, *Paraphlebotomus* Theodor and *Adlerius* Nitzulescu were uninfected. The infection rate was 20.59% in *Ph. papatasi*, the only representative of the subgenus *Phlebotomus* Rondani and Berte*.* Within the *Larroussius* Nitzulescu subgenus, only *Ph. major* s.l. (62.74%) and *Ph. tobbi* (0.83%) tested positive, while all other species were negative. In addition, one *Ph. economidesi* specimen (subgenus *Transphlebotomus* Adler) and *Se. minuta* (3.03%, subgenus *Sergentomyia* França and Parrot) were found to be infected, whereas other members of these subgenera tested negative. The variation in infection rates among the sand fly taxa identified in this study aligns with previous research, which has shown that *Wolbachia* prevalence can vary by species and geographic location. For instance, the overall infection rate in Moroccan sand flies was low compared with that recorded in Spanish populations. In Spain, infection rates varied significantly with both altitude and species, whereas no such associations were observed in Morocco [20]. Focusing on *Ph. sergenti*, all specimens collected from Türkiye were uninfected, similar to findings from Spain [20]. The same species in Morocco and Iran, however, exhibited a *Wolbachia* prevalence of around 20% and 70%, respectively [20, 43].Variation in *Wolbachia* infection rates among sand fly species has also been documented in New World taxa [44–47].

The microbial community structure of arthropods, including vectors, is shaped by multiple interacting factors such as host phylogeny, temperature, habitat characteristics, pathogen infection status and physiological condition of the host [48–51]. Therefore, it is not possible to provide a definitive explanation for the high *Wolbachia* infection rate observed in *Ph. major* s.l., as opposed to the absence or very low prevalence in other taxa. Host phylogeny may be ruled out as a primary explanatory factor, as closely related taxa within the same subgenus, such as *Ph. kandelakii* s.l. and *Ph. perfiliewi* s.l., were entirely uninfected in our study, and *Ph. tobbi* was represented by only a single infected female specimen. This contrasts with our findings in *Ph. major* s.l., where infection rates were markedly high. The only previous study assessing *Wolbachia* infection in these sand fly taxa, conducted in Iran, reported no infection in *Ph. major* and *Ph. tobbi*, but detected low infection rates of approximately 2% and 22% in *Ph. kandelakii* and *Ph. perfiliewi*, respectively [52]. Local environmental conditions are also unlikely to account for the observed differences, as most of these taxa were collected from the same sampling sites, and in many cases, from the very same traps (in our case) (Additional file 2). The limited number of specimens belonging to certain taxa (i.e. *Ph. caucasicus* Marzinovsky, 1917 and *Ph. brevis* Theodor and Mesghali, 1964)*,* collected from a restricted number of sampling points should also be taken into consideration. Nevertheless, the variation in infection rates observed may be associated with differences in the hosts’ local genetic background influencing their competence for infection and/or historical infection dynamics.

The marked mitochondrial divergence previously documented in several taxa from Türkiye – irrespective of geographical origin, as several divergent lineages were found in sympatry or collected from nearby locations – prompted us to investigate whether *Wolbachia* infection might underlie or contribute to this divergence. Except for *Ph. major* s.l., all other taxa tested negative for *Wolbachia* infection, suggesting that alternative mechanisms may be responsible for the observed mitochondrial diversification. The *Ph. major* species complex, whose members are largely allopatric, comprises six formally described species (*Ph. neglectus, Ph. notus, Ph. major, Ph. syriacus, Ph. wenyoni* and *Ph. wui*). Previous work has indicated that this complex has a relatively wide distribution in Türkiye. Based on mitochondrial *cox1* data, this study identified *Ph. neglectus, Ph. syriacus,* and three additional, formally undescribed lineages. *Ph. neglectus* is primarily found in the easternmost and westernmost regions, whereas *Ph. syriacus* is restricted to southeastern regions; both species occur sympatrically in some southeastern localities. The third undescribed lineage occurs in southeastern and northeastern regions, the fourth lineage is found only in northeastern regions, and the fifth mitochondrial lineage has the broadest distribution, co-occurring with other lineages in certain localities [25]. Consistent with previous findings, species delimitation analyses (TCS and ASAP) based on mitochondrial *cox1* data – including both historical and recently collected specimens – confirmed the presence of five distinct lineages within this species complex. Supporting our hypothesis, logistic regression analysis revealed a significantly higher probability of *Wolbachia* infection in the undescribed lineages of the complex compared with the formally described *Ph. neglectus* and *Ph. syriacus*, with no significant association with sex. Furthermore, comparisons of specimens collected at the same sampling sites across different time periods indicated a consistent infection status within these lineages. However, collecting additional temporal data across the remaining lineages of *Ph. major* s.l. would strengthen conclusions regarding the stability and prevalence of *Wolbachia* within the entire species complex.

The ML analyses based on both *wsp* and MLST datasets were conducted to evaluate whether different sand fly lineages or taxa were infected with distinct *Wolbachia* supergroups or strains. All infected specimens were assigned to Supergroup A. Two unique *wsp* haplotypes were detected across all tested taxa, neither of which matched any *Wolbachia* sequences previously reported from other sand flies in public databases. One of these haplotypes was shared by multiple taxa – *Ph. tobbi*, *Ph. papatasi*, *Se. minuta*, and the various lineages of *Ph. major* s.l. – while *Ph. economidesi* had the second, unique haplotype. Phylogenetic analysis based on MLST data corroborated the Supergroup A assignment and revealed unique allelic profiles for *Ph. economidesi*, one *Ph. papatasi* specimen, one infected *Ph. neglectus* specimen (lineage 1) and lineage 4. In contrast, lineages 3 and 5, along with another *Ph. papatasi* specimen, shared an identical MLST profile. Among the divergent profiles, *Ph. economidesi* and the second *Ph. papatasi* specimen exhibited entirely distinct allelic combinations. While *Ph. economidesi* clustered with the other infected sand fly taxa and a *Sergentomyia* species – suggesting a close phylogenetic relationship – the divergent *Ph. papatasi* specimen grouped with *Lutzomyia* species, indicating a phylogenetically distinct *Wolbachia* strain. *Wolbachia* infections with identical strains in different sand fly species have been reported in previous studies based on the *wsp* marker, suggesting possible horizontal transmission [20, 52]. This is plausible given that these species share the same habitats during both their pre-adult and adult life stages, potentially facilitating the transfer of microbial agents. However, as relying solely on either the *wsp* or MLST dataset may lead to misinterpretations – since the two approaches can yield differing estimates of relatedness among strains [31] – further confirmation using multilocus sequence typing would help determine whether the infections are caused by the same *Wolbachia* strain. Given that MLST amplification failed in some taxa (*e.g. Ph. tobbi* and *Se. minuta*), not all *Wolbachia*-positive *Ph. major s.l.* lineage 4 specimens yielded complete allelic profiles, inferences regarding strain diversity and potential horizontal transmission should be considered preliminary until full MLST profiles can be recovered.

The shared cytoplasmic inheritance of arthropod mitochondria and *Wolbachia* may result in a selective sweep on host mitochondria, similar to the hitchhiking effect of an advantageous mutation in a population, depending on the impact of *Wolbachia* on host fitness. This sweep in turn, reduces the mitochondrial variation and a deviation from neutrality [53]. The widely distributed *Wolbachia* infected lineage 5 of *Ph. major* s.l. exhibited signs of such a sweep, with reduced nucleotide diversity and significantly negative neutrality statistics, compared with the mostly uninfected lineage 1 (*Ph. neglectus*) and completely uninfected lineage 2 (*Ph. syriacus*). ASAP and TCS network analyses support the divergence of these lineages, indicative of an effect of *Wolbachia* infection. The significantly negative values of Tajima’s D and Fu’s Fs in uninfected lineages (1 and 2), together with their relatively high haplotype and nucleotide diversity, suggest a signal of historical population expansion. Given that the speciation within this complex was estimated to date back to the early Pleistocene, which coincided with the glacial and interglacial periods [54], post-glacial expansion within these lineages, as seen in several other sand fly taxa [55–57], appears to be a more plausible explanation for the observed neutrality test results. Although our sampling spans multiple years (2005–2016, with additional collections in 2023), the temporal range is insufficient to account for the observed mitochondrial divergence, given the substitution rate of the *cox1* gene [54]. Furthermore, specimens collected from the same localities in different years (e.g. Adana and Ankara) consistently clustered within the same lineages, indicating temporal stability rather than divergence. Spatial separation is also unlikely to be the sole driver, as several divergent lineages were detected in sympatry. Accordingly, while demographic history may have contributed to some of the observed structure, *Wolbachia*-associated divergence remains the most parsimonious explanation. However, we were unable to draw robust conclusions regarding the impact of *Wolbachia* infection on the mitochondrial diversity of lineages 3 and 4, owing to their limited sample sizes; addressing this would require additional specimens and experimental crossing studies. In addition, the potential effects of different *Wolbachia* sequence types – considering the lineages 3 and 5 are infected with the same strain, while lineage 4 harbours a distinct one – could not be fully assessed, as only a limited number of complete MLST allelic profiles were obtained for each lineage. Nevertheless, the absence of recombination among MLST loci suggests that the *Wolbachia* strains infecting *Ph. major* s.l. lineages are likely clonal [31].

The impact of *Wolbachia* on mitochondrial diversity has been demonstrated in several insect taxa, revealing morphotype-specific strains in the fruit fly *Anastrepha fraterculus* (Wiedemann, 1830) complex [21], acting as a reinforcing factor in the speciation process of the weevil *Pantomorus postfasciatus* (Hustache, 1947) [58], and indicating a potential role of the infection in restricting gene flow between members of the fig wasp *Wiebesia pumilae* [23]. Moreover, by analysing a comprehensive dataset comparing infected and uninfected closely related arthropod taxa, Cariou et al. (2017) showed that *Wolbachia* infections are associated with reduced mitochondrial diversity [59]. To the best of our knowledge, evidence on the impact of *Wolbachia* infection on sand fly diversification is very limited. Supporting our findings, Azpurua et al. (2010) detected a highly divergent *Lutzomyia vespertilionis* (Fairchild and Hertig 1947) specimen compared with its conspecifics based on *cox1* data analysis [44]. By comparing MLST allelic profiles obtained for *Lu. vespertilionis* specimens and its divergent form, they speculated that the different *Wolbachia* strains detected may be responsible for this diversification, and noted that further investigation involving a larger number of specimens is needed to clarify the effect of incompatible strains on sand fly divergence. More recently, Torres-Llamas et al. (2025) highlighted the potential role of *Wolbachia* infection in shaping genetic structure and its epidemiological relevance for cutaneous leishmaniasis in *Ph. sergenti* from Morocco [20]. Although they did not conduct a direct analysis of mitochondrial lineages, they reported an apparent association between *wsp* haplotypes, predicted WSP protein structures, and previously described mitochondrial clades – suggesting a possible link that needs further investigation.

## Conclusions

Although sand fly microbiota are increasingly recognised for their roles in vector fitness [60, 61] and pathogen development [50, 62, 63], the presence and potential effects of *Wolbachia* – a natural component of the microbiota in some species – require further investigation [64, 65]. In this study, we retrospectively assessed natural *Wolbachia* infection in sand flies collected across a broad geographical and temporal range in Türkiye and explored its potential contribution to mitochondrial divergence in selected taxa. *Wolbachia* was detected in five taxa with varying prevalence, and five distinct strains were identified. The highest prevalence occurred in *Ph. major* s.l., where evidence supported *Wolbachia*-associated mitochondrial divergence within this complex. Infection estimates for taxa represented by few specimens should be interpreted cautiously, and expanded sampling across their distribution ranges is required. MLST profiles should also be completed for *Wolbachia*-positive species lacking strain-level data to refine prevalence estimates and characterise circulating *Wolbachia* diversity in Türkiye. Complementary approaches, such as whole-genome sequencing, could also be applied in future studies to further resolve *Wolbachia* strain diversity. Further analyses using broader nucleotide datasets, alongside crossing experiments, are needed to assess *Wolbachia*-mediated gene flow restriction between lineages, particularly given their sympatric distributions. In addition, laboratory and field-based studies on *Wolbachia–Leishmania* interactions would further support sand fly-borne disease management efforts.

## Supplementary Information

Additional file1 (XLS 31 kb)

Additional file2 (XLS 17 kb)

Additional file3 (XLS 21 kb)

Additional file4 (PDF 560 kb)

## Data Availability

The sequence data generated in this study will be made publicly available in GenBank upon acceptance of the manuscript for publication.

## Abbreviations

WHO: World Health Organization
NTDs: Neglected tropical diseases
VL: Visceral Leishmaniasis
CL: Cutaneous Leishmaniasis
CI: Cytoplasmic incompatibility
SFBDs: Sand fly-borne diseases
MLST: Multi-locus sequence typing
ML: Maximum likelihood
ASAP: Assemble species by automatic partitioning
TCS: Templeton–Crandall–Sing network
OR: Odds ratios
CI: Confidence interval
ST: Sequence type
HD: Haplotype diversity
