# Supplementary figures and images for: Prevalence of Wolbachia in natural sand fly (diptera: psychodidae) populations from Türkiye and its potential role in mitochondrial divergence
Source: Parasit Vectors. 2025 Dec 4;19:16. doi: 10.1186/s13071-025-07157-4 (PMC12797604; doi:10.1186/s13071-025-07157-4)

A

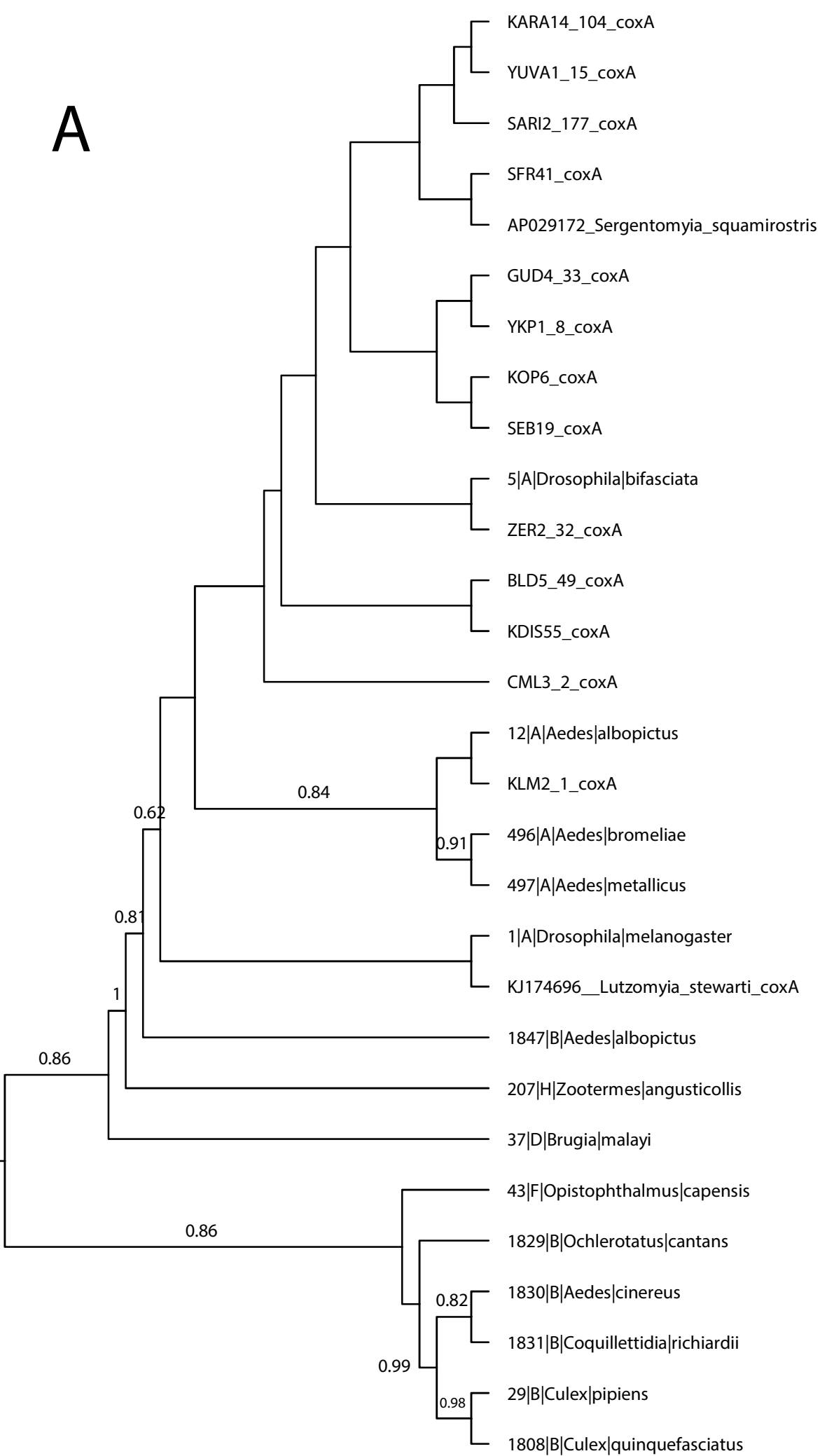

B

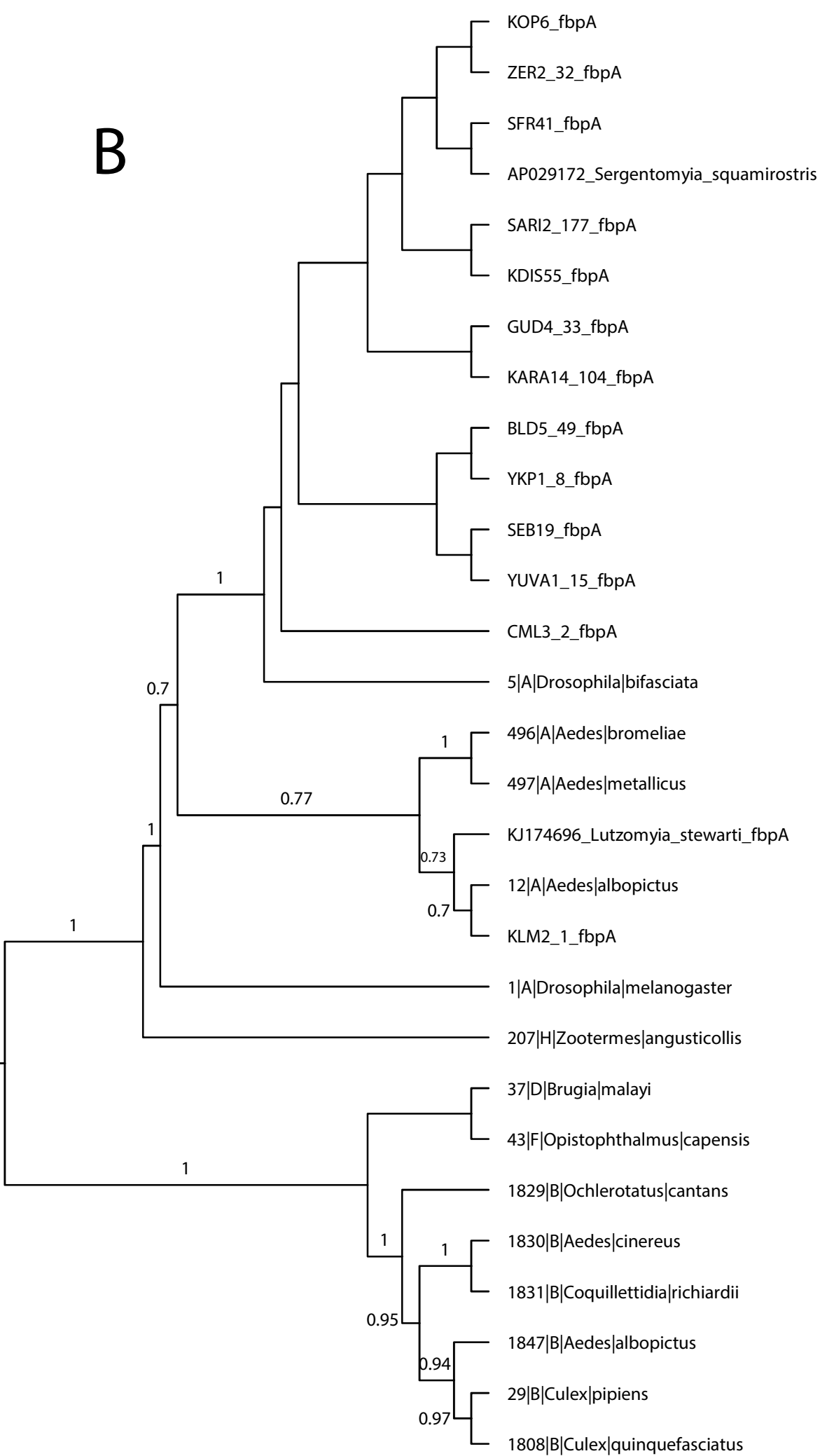

C

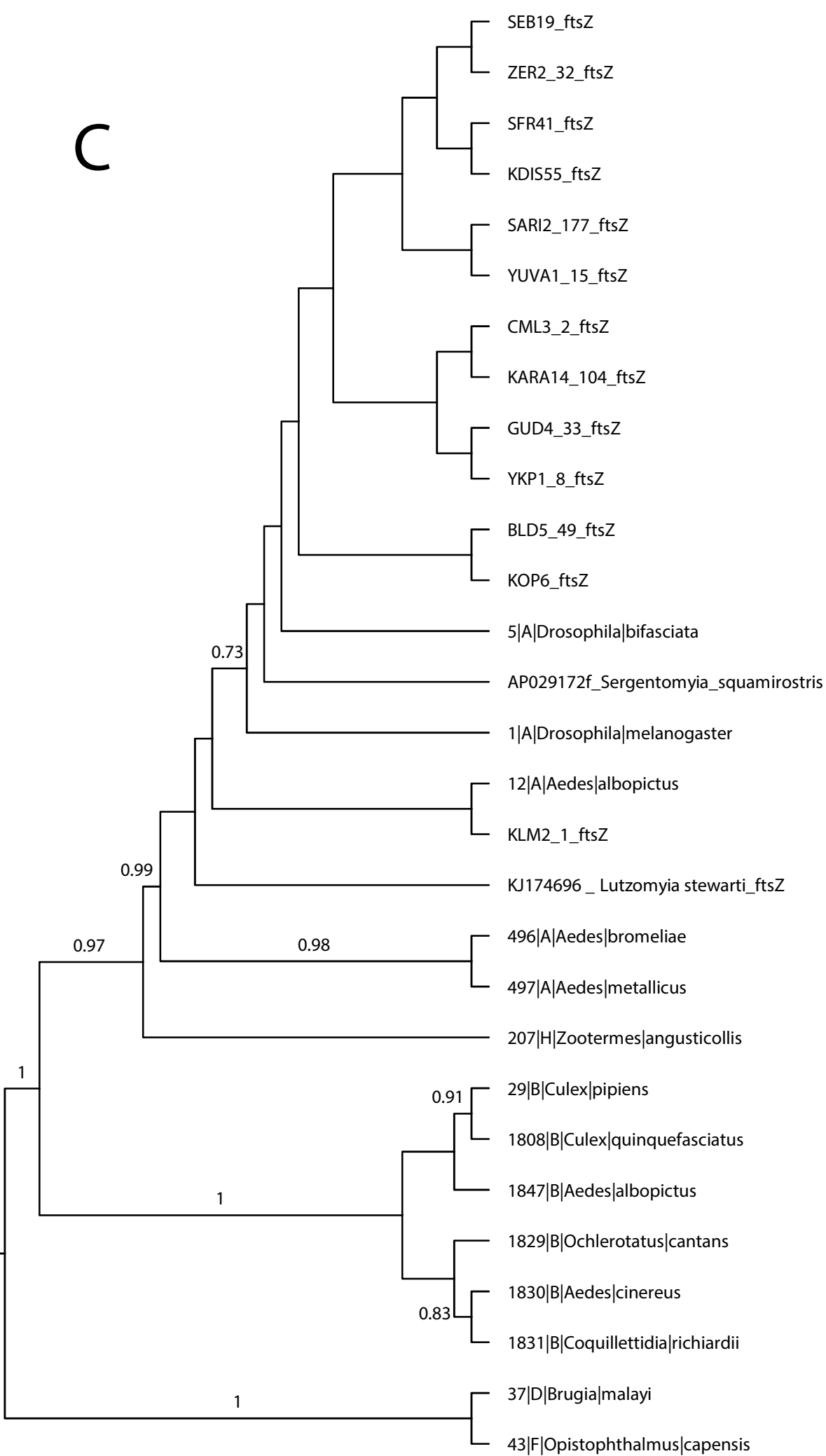

D

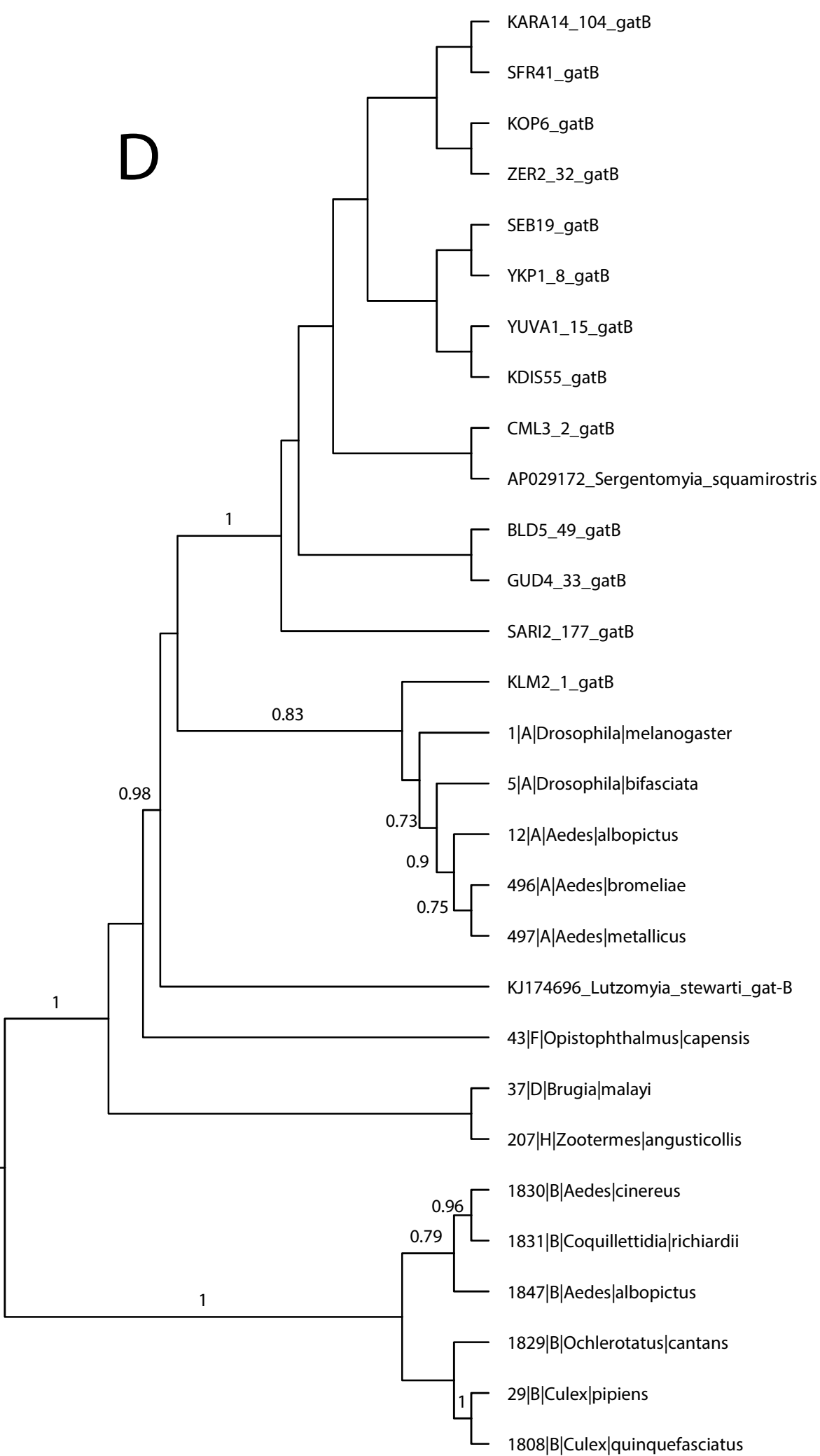

E

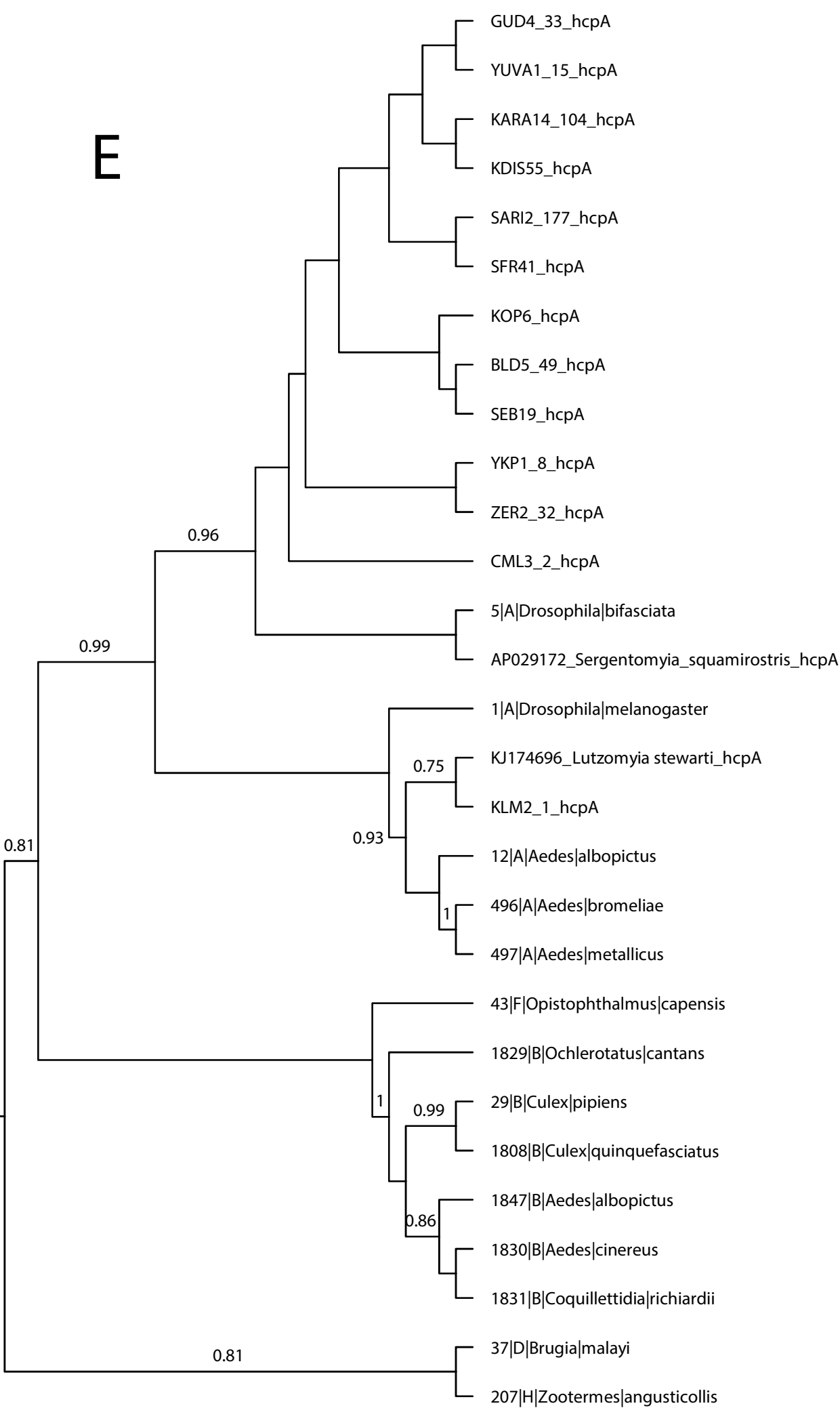

Supplement: Supplementary file 4 — Additional file4 (PDF 560 kb) [file 13071_2025_7157_MOESM4_ESM.pdf]
